# The Evaluation of Variations in Patterns of Sphenoid Sinus Pneumatization Using Computed Tomography in a South Indian Population

**DOI:** 10.7759/cureus.23174

**Published:** 2022-03-15

**Authors:** B H Parameshwar Keerthi, Shivaprasad G Savagave, Anil K Sakalecha, Vineela Reddy, Yashas Ullas L

**Affiliations:** 1 Radiology, Sri Devaraj Urs Academy of Higher Education and Research, Kolar, IND; 2 Radiodiagnosis, Sri Devaraj Urs Academy of Higher Education and Research, Kolar, IND

**Keywords:** paranasal sinuses, clivus, lesser wing of sphenoid, pterygoid plates, pre-sellar, sellar, pneumatization, sphenoid sinus, computed tomography

## Abstract

Background and objective

Knowledge about sphenoid sinus pneumatization is critical for skull base surgeries and functional endoscopic sinus surgery (FESS) in order to avoid serious complications like postoperative meningitis, sinusitis, cerebrospinal fluid (CSF) rhinorrhea, and intracranial hematoma. In this study, we aimed to assess the proportion of anatomical variants in sphenoid sinus pneumatization and to determine the common sphenoid pneumatization pattern in a South Indian population.

Methods

This retrospective study was conducted over a period of six months from July 2019 to December 2019 among 573 patients who underwent non-contrast CT (NCCT) or contrast-enhanced CT (CECT) of the brain, paranasal sinuses (PNS), orbit, and face.

Results

Most of the patients were in the age group of 20-39 years. The male-to-female ratio was 2.45:1. Among the posterior extensions, the most common variant was type D, followed by type C, type B, and type A. Among the clival extensions, the most common variant was Cliv-A, followed by Cliv-B, Cliv-C, and Cliv-D. The most common lateral wall pneumatization was bilateral lateral wall pneumatization followed by unilateral sinus wall pneumatization. Lat-A was the most common lateral wall pneumatization pattern followed by Lat-D, Lat-B, and Lat-C.

Conclusion

Our study intends to classify the sphenoid sinus pneumatization pattern and identify the most common variant among them, thereby guiding the skull base and FESS surgeons in choosing the correct mode of the operative procedure and also anticipating and avoiding complications of surgery.

## Introduction

The sphenoid bone is a complex structure located at the center of the skull base. It is bounded anteriorly by the frontal and ethmoidal bones, laterally by the temporal bone, and posteriorly by the occipital bone. It represents a transition between the intracranial and extracranial components of the skull [1]. Clinically, the assessment of the sphenoid sinus is difficult when compared to other paranasal sinuses (PNS). Furthermore, many complex neurovascular structures are located adjacent to the sphenoid sinus [2,3]. The sphenoid sinus is the last sinus to be pneumatized, which begins at birth and is completed at age of 12 years. Knowledge about sphenoid sinus pneumatization is important for radiologists and surgeons to assess the transsphenoidal approach for the resection of pituitary tumors and to perform functional endoscopic sinus surgery (FESS) [2]. A transsphenoidal approach without the knowledge of sphenoid sinus pneumatization can lead to serious complications like injury to major cranial nerves or arteries such as middle meningeal and vidian arteries and cavernous sinus thrombosis [4]. This study aims to explore the various patterns of pneumatization in the sphenoid sinus and their prevalence in the South Indian population, which will provide guidance to surgeons in predicting and minimizing intraoperative complications.

## Materials and methods

This retrospective study was conducted among patients referred to the Department of Diagnostic Radiology who underwent either non-contrast CT (NCCT) or contrast-enhanced CT (CECT) of the brain, PNS, orbit, or face from July 2019 to December 2019. Patients with fractures of the sphenoid bone and those with head and neck malignancies eroding the sphenoid bone were excluded from the study. CT was performed using the Siemens Somatom Emotion 16 slice CT system (Siemens Healthineers AG, Erlangen, Germany). In these patients, the CT pattern of pneumatization was recorded by a single radiologist.

Classification of the sphenoid sinus

**Figure 1:**
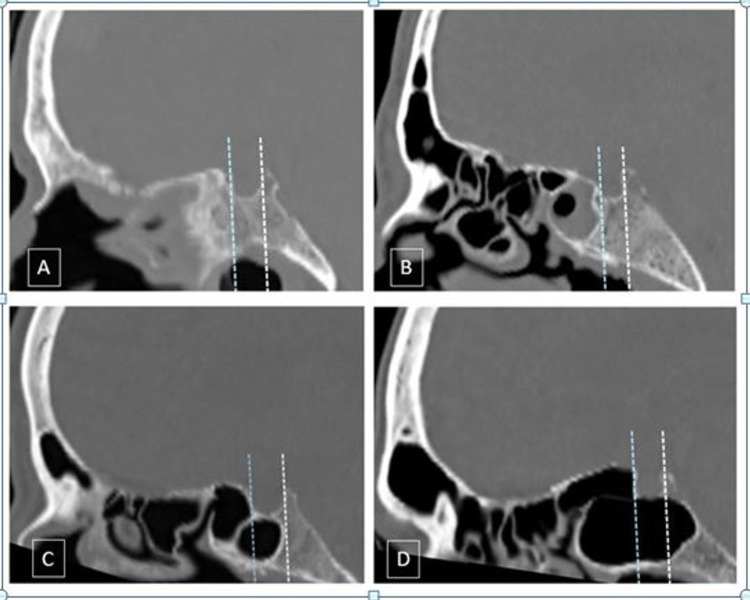
Classification of the posterior extent of sellar pneumatization in the sphenoid sinus CT bone window midsagittal sections showing the various types of sphenoid pneumatization. A: Conchal type; pneumatization (>10 mm) anterior to the anterior wall of sella. B: Presellar type; posterior margin of pneumatization anterior to the anterior wall of the sella. C: Incomplete sellar; posterior margin of pneumatization posterior to the anterior wall but anterior to the posterior wall of the sella. D: Complete sellar; pneumatization extends posteriorly to the posterior wall of the sella CT: computed tomography

**Figure 2:**
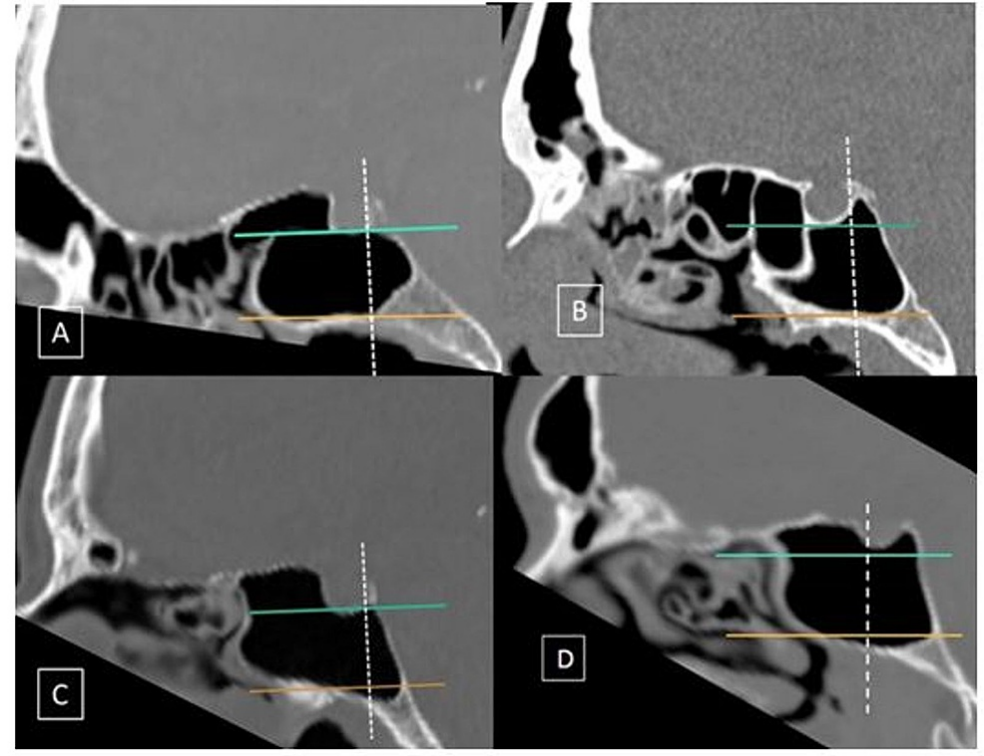
Classification of craniocaudal extension of sellar pneumatization in the sphenoid sinus CT bone window mid-sagittal sections showing the various types of sphenoid pneumatization. Horizontal lines are drawn along the inferior margin of the sella (green line) and the vidian canal (orange line). The vertical line is along the posterior wall of the sella (dotted white line). A: Pneumatization does not extend above the inferior margin of the sella or below the level of the vidian canal. B: Dorsal; pneumatization extending into the dorsum sella. C: Occipital; pneumatization that extends inferiorly to the vidian canal level. D: Combined; dorsal + occipital CT: computed tomography

**Figure 3:**
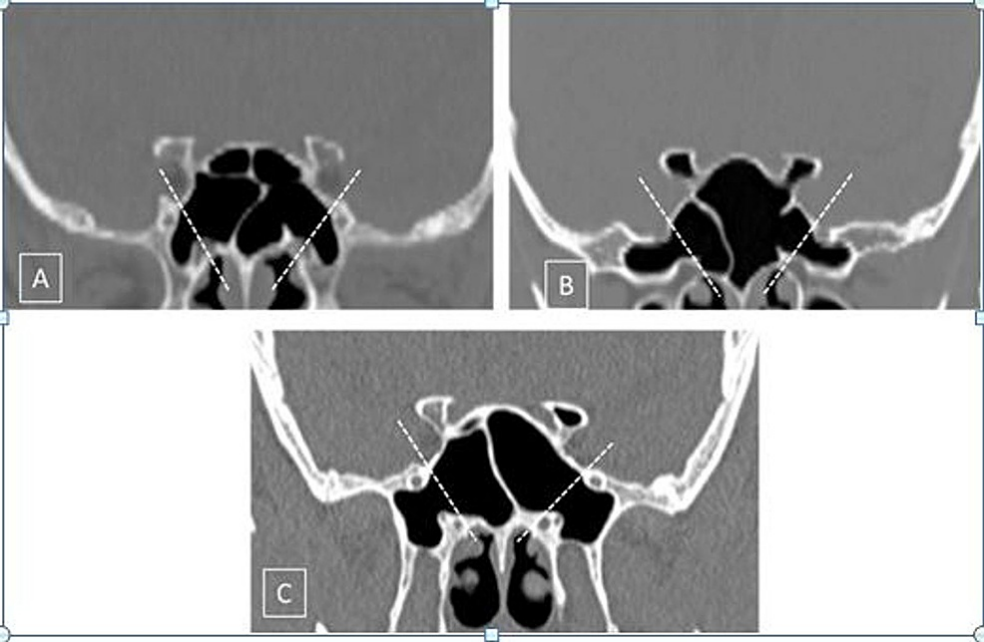
Lateral and lesser wing pneumatization of the sphenoid sinus Two lines are drawn along the medial margins of the foramen rotundum and the vidian canal (white dotted lines) on both sides. A: Pterygoid; pneumatization on both sides inferior to the vidian canal extending into the pterygoid process. B: Greater wing of the sphenoid; pneumatization extending laterally into the greater wing of sphenoid on both sides, beyond the foramen rotundum. Lesser wing; pneumatization extending into the bilateral anterior clinoid processes. C: Full lateral; pterygoid + lateral on both sides. Also noted is the lesser wing extension on the left side

There are four types of sphenoidal sinuses: A, B, C, and D. Type A is the conchal type, type B is the presellar type, and types C and D are classified as incomplete and complete sellar types (Figure 1). Additionally, the extension of pneumatization to the clivus is classified into four patterns: Cliv-A to Cliv-D. Clival extension of sphenoid sinus pneumatization is assessed by drawing three imaginary lines: two horizontal lines (one along the floor of the sella and another line corresponding to the vidian canal) and one vertical line drawn along the posterior wall of the sella. Cliv-A is a subdorsal type in which pneumatization does not extend above the level of the inferior margin of the sella. Cliv-B is the dorsal type that indicates superior extension of pneumatization into the dorsum sella. Cliv-C is an occipital type where the pneumatization extends inferiorly to the vidian canal. Cliv-D is a combination of Cliv-B and Cliv-C (Figure 2). Pneumatization of the sphenoid sinus can extend laterally to include the pterygoid plate and the greater wing or lesser wings of the sphenoid sinus. The lateral extension is classified into four categories: Lat-A, Lat-B, Lat-C, and Lat-D. Lat-A indicates lateral extension to include the pterygoid process. Lat-B indicates extension into the greater wing of the sphenoid. Lat-C is the combination of Lat A and B. Lat-D indicates extension of sphenoid sinus pneumatization into the lesser wing of the sphenoid (Figure 3) [4].

Statistical analysis

The data were entered into a Microsoft Excel sheet. The measurable variables were analyzed and interpreted by the student’s t-test and the ordinal and categorical variables between them were interpreted by the Chi-squared (χ2) test. The statistical analyses were performed using the SPSS Statistics software version 21 (IBM, Armonk, NY) and OpenEpi version 3.01.

## Results

The study included 573 patients, of which 71% (n = 407) of patients were males and 29% (n = 166) were females (male-to-female ratio of 2.45:1). The majority of the patients were aged between 20 and 39 years (n = 225; 39.27%), followed by those aged between 40 and 59 years (n = 198; 34.55%). Patients aged <20 years constituted only 2.27% of the study population (n = 13). The distribution of males and females was similar across all the age groups (p>0.5; not significant) (Table 1).

**Table 1:** Gender and age distribution P>0.5; not significant

Age (years)	Gender		Total	%
	Male, n (%)	Female, n (%)		
<20	7 (1.22%)	6 (1.05%)	13	2.27
20-39	164 (28.62%)	61 (10.65%)	225	39.27
40-59	143 (24.96%)	55 (9.6%)	198	34.55
60-79	72 (12.57%)	35 (6.11%)	107	18.67
80-100	22 (3.84%)	8 (1.4%)	30	5.24
Total	407 (71%)	166 (29%)	573	100

The sellar type of the sphenoid sinus (types C and D) constituted the most number of cases in the population (n = 504; 88%). Type D was the most common type and was seen in 55.5% (n = 318) of patients, followed by type C in 32.46% (n = 186), and type B in 10.47% (n = 60) of the study population. Type A was seen in the least number of patients (n = 9; 1.57%).

In 318 patients with clival pneumatization, the majority of the patients had the type Cliv-A (subdorsal) type of clival extension (n = 214; 67.32%), followed by dorsal, occipital, and combined forms, which constituted 21.38% (n = 68), 5.97% (n = 19), and 5.34% (n = 17) of patients respectively (Table 2).

**Table 2:** Classification based on clival pneumatization

Clival	Gender		
	Males	Females	Total
No pneumatization	170	85	255
A	154	60	214
B	56	12	68
C	12	7	19
D	15	2	17
Total	407	166	573

The lateral extension indicates pneumatization extending to the pterygoid and greater and lesser wings of the sphenoid. Based on this categorization, four variants were derived: Lat-A to Lat-D. Lat-A represents pneumatization that extends to the pterygoid plates and Lat-B denotes extension into the greater wing of the sphenoid. Lat-C is defined as the lateral extension beyond pterygoid plates, and Lat-D denotes extension into the lesser wing of the sphenoid. In our study, 1146 sinus walls were assessed for lateral extensions. The lateral extension is further divided into three types: bilateral symmetrical lateral pneumatization, bilateral asymmetric lateral pneumatization, and unilateral pneumatization. Symmetrical pneumatization represents a similar type of lateral pattern on both sides whereas asymmetric indicates a different pattern of lateral pneumatization on the right and left sides [5]. Among 1146 sinus walls assessed, 477 (41.62%) showed lateral pneumatization and 669 (58.38%) sinus walls did not. Among 477 instances of lateral pneumatization, 237 (20.68%) were unilateral and 162 were bilaterally similar (14.13%) (any of Lat-A to Lat-D). In the remaining 78 (6.8%) sinus walls, there was asymmetrical lateral pneumatization between the right and left sides.

In 162 cases of bilaterally symmetrical lateral pneumatization, Lat-A was seen in 48 cases (29.63%), Lat-B in 44 cases (27.16%), Lat-C in 30 (18.52%), and Lat-D in 40 cases (24.7%). Among 78 bilateral asymmetrical variants of lateral pneumatization, Lat-A was seen in 11 cases on the right side and in 10 cases on the left side. Lat-B was seen in six and seven cases on the right and left sides respectively. Lat-C was present in four cases on the right side and 10 cases on the left side. Lat-D was seen in 18 cases on the right and 12 cases on the left side. Overall, there were 78 cases of bilaterally asymmetrical lateral pneumatization of which Lat-A constituted 26.92% (n = 21), Lat-B constituted 19.23% (n = 15), Lat-C constituted 17.95% of the cases (n = 14), and Lat-D constituted 38.46% (n = 30). Among 237 cases of unilateral lateral pneumatization, Lat-A constituted 30.8% (n = 73) of cases. Lat-B constituted 29.95% (n = 71) of cases. Lat-C was seen in 40 (16.88%) and Lat-D was seen in 53 cases (22.36%). In 477 cases with lateral pneumatization, Lat-A was seen in 142 cases (29.77%), Lat-B was seen in 128 cases (26.83%), Lat-C was seen in 84 cases (17.61%), and Lat-D was seen in 123 cases (25.79%) (Table 3).

**Table 3:** Classification based on lateral pneumatization

Bilateral symmetrical (n = 162), n (%)	Bilateral asymmetrical (n = 78), n (%)		Unilateral (n = 237), n (%)	Total, n (%)
	Right	Left		
A = 48 (10%)	A = 11 (2.3%)	A = 10 (2.1%)	A = 73 (15.3%)	A = 142 (29.7%)
B = 44 (9.3%)	B = 6 (1.2%)	B = 7 (1.5%)	B = 71 (14.8%)	B = 128 (26.8%)
C = 30 (6.3%)	C = 4 (0.9%)	C = 10 (2.1%)	C = 40 (8.3%)	C = 84 (17.6%)
D = 40 (8.4%)	D = 18 (3.7%)	D = 12 (2.5%)	D = 53 (11.1%)	D = 123 (25.7%)
162 (33.9%)	39 (8.1%)	39 (8.1%)	237 (49.9%)	477 (100%)

## Discussion

The role of imaging has expanded in the evaluation of the sphenoid sinus as part of the preoperative assessment. The importance of the variability in pneumatization and its impact on surgery is well known. Hence, it is critical that radiologists are aware of the patterns of pneumatization of the sphenoid sinus [6]. Based on pneumatization patterns, Hammer and Radberg classified the sphenoid sinus into three types: conchal, presellar, and sellar. In their study, they found that the sellar variant was the most common pattern, constituting 85% of the total cases, followed by presellar and conchal variants, which constituted 11% and 2.5% of cases respectively [7]. Furthermore, Hamid et al. added a new subgroup to the sphenoid sinus, which is the post-sellar variant [8]. The post-sellar variant was named the "complete sellar variant" by Hiremath. Cerebrospinal fluid (CSF) rhinorrhea is more common in patients who have a post-sellar variant. Because post-sellar variants are more common, it is critical to report their presence to avoid the risk of post-surgical CSF rhinorrhea [4]. It has been reported in the literature that there is a significant difference between the patterns of sphenoid sinus pneumatization [8-10]. The observations from our study were statistically different from those reported in the literature and showed variations between people of different ethnicity, race, and regions. This study's findings differ from the observations by Hiremath et al., thereby reiterating the importance of classifying pneumatization and its impact on surgery based on specific local populations.

Studies among various ethnicities conducted by different researchers have shown that the subdorsal type constitutes the majority of the clival pneumatization variant [4,5,10]. A similar observation was made in our study. There was a statistically significant difference in the clival pneumatization pattern between our study and other studies. Transsphenoidal surgery is a minimally invasive procedure that provides access to the entire length of clivus from dorsum sella cranially to foramen magnum caudally. The inferior wall of the sphenoid sinus divides the clivus into the upper sphenoid portion and the lower rhino-pharyngeal portion [11]. The clival extension of the sphenoid sinus is more suitable for the transnasal entry into the posterior cranial fossa when compared to the non-pneumatized clivus. The average clival thickness in clival extension of sphenoid pneumatization is 3.2 mm [5]. The transnasal approach is more suitable for performing biopsies and resections in clival chordoma and other craniovertebral junction lesions. If pneumatization is extensive, there is a greater chance of post-procedure CSF rhinorrhoea occurring, because of the risk of bony dehiscence and protrusion of neurovascular structures into the sinus [4]. The sphenoid portion of the clivus is closely related to the dural layer of brainstem structures. The carotid protuberance, sixth cranial nerve, and dorsal meningeal artery are located medial to the paraclival carotid artery, and damage to these structures in the transclival approach is more common. The lower part of the clivus is closely associated with the foramen of the lacerum. Hence, the lacerus part of the internal carotid artery is also vulnerable to damage in the transclival approach [12].

Vidian canal and foramen rotundum are two important landmarks in FESS surgery and the line joining them is termed the VR line. Extension of pneumatization up to the vidian canal and foramen rotundum is important to avoid injury to them. Lat-A to Lat-C indicate pneumatization extending up to the vidian canal and foramen rotundum. Lat-D represents lesser wing pneumatization that does not involve VR line and they are prone to injury. Pterygoid process pneumatization can occur alone (Lat-A) or in association with the greater wing of sphenoid (Lat-C). Pterygoid process pneumatization results in the thinning of the roof of the scaphoid fossa, which leads to a thin dividing plate between the sphenoid sinus and the auditory tube. Damage to the roof of the scaphoid fossa during surgery may result in abnormal communication between the sphenoid sinus and auditory tube [10]. If the tympanic membrane is intact, this will present as CSF rhinorrhoea and if the tympanic membrane is perforated, it presents as CSF otorrhea. In our study, Lat-A and C together constituted 226 (44.47%) cases. These patients are more vulnerable to developing CSF otorrhea [13]. Pneumatization of the greater wing of the sphenoid can be alone (Lat B) or in combination with pterygoid pneumatization (Lat C). Although pneumatization of the greater wing of the sphenoid in itself will not cause any significant complication, the presence of arachnoid granulation along the inner edge of the greater wing of the sphenoid can lead to iatrogenic CSF rhinorrhoea [12].

Various studies on sphenoid sinus pneumatization have shown an association of the lesser wing of sphenoid and anterior clinoid process pneumatization with post-surgical optic nerve and internal carotid artery trauma. In our study, we categorized Lat-D patterns for pneumatization of the lesser wing of the sphenoid and anterior clinoid process. The optic nerve and ophthalmic branch of the internal carotid artery are in close proximity to the lateral wall of the sphenoid sinus. In the optic canal, the optic nerve is least nourished and is the most susceptible part to get injured. The optic canal is formed by the lesser wing of the sphenoid. It transmits vital structures such as the optic nerve and ophthalmic artery. The intracranial segment of the optic nerve is vulnerable to damage during transsphenoidal surgeries. The internal carotid artery lies lateral to the optic nerve [9]. A study conducted by Hewaidi et al. showed that anterior clinoid process pneumatization would form an opticocarotid recess, which is a small space on the lateral wall of the sphenoid sinus, between the optic canal and the carotid prominence. The optic nerve and internal carotid artery will occupy the recess. In anterior clinoid process pneumatization, the protrusion of the optic nerve and internal carotid artery can be seen. The study by Birsen et al. revealed 24.1% anterior clinoid process pneumatization in the Turkish population [14], which is similar to our findings. In these variants, sphenoid sinus must be accessed by opening the anterior and posterior ethmoids so that the optic nerve is medial and inferior, and hence the optic nerve penetration and injury to the internal carotid artery can be avoided [10,15].

## Conclusions

Based on our findings, we conclude that the major extensions of the sphenoid sinus are posterior, lateral, and posteroinferior extensions. We analyzed a large group of patients belonging to a South Indian population and found that the complete sellar variant, subdorsal variant, and pterygoid pneumatization were the most common variant in posterior, posteroinferior, and lateral sphenoid sinus extensions respectively. We believe this study will help radiologists in identifying the different variants of sphenoid sinus pneumatization and thereby guide otorhinolaryngologists as well as FESS and skull base surgeons with regard to optimal decision-making in minimally invasive surgeries.

## References

[1] Chong VF, Fan YF, Tng CH (1998). Pictorial review: radiology of the sphenoid bone. Clin Radiol.

[2] Wiebracht ND, Zimmer LA (2014). Complex anatomy of the sphenoid sinus: a radiographic study and literature review. J Neurol Surg B Skull Base.

[3] Gibelli D, Cellina M, Gibelli S, Oliva AG, Termine G, Sforza C (2017). Anatomical variants of sphenoid sinuses pneumatisation: a CT scan study on a Northern Italian population. Radiol Med.

[4] Hiremath SB, Gautam AA, Sheeja K, Benjamin G (2018). Assessment of variations in sphenoid sinus pneumatization in Indian population: a multidetector computed tomography study. Indian J Radiol Imaging.

[5] Wang J, Bidari S, Inoue K, Yang H, Rhoton A Jr (2010). Extensions of the sphenoid sinus: a new classification. Neurosurgery.

[6] Güldner C, Pistorius SM, Diogo I, Bien S, Sesterhenn A, Werner JA (2012). Analysis of pneumatization and neurovascular structures of the sphenoid sinus using cone-beam tomography (CBT). Acta Radiol.

[7] Hammer G, Radberg C (1961). The sphenoidal sinus. An anatomical and roentgenologic study with reference to transsphenoid hypophysectomy. Acta radiol.

[8] Hamid O, El Fiky L, Hassan O, Kotb A, El Fiky S (2008). Anatomic variations of the sphenoid sinus and their impact on trans-sphenoid pituitary surgery. Skull Base.

[9] Anusha B, Baharudin A, Philip R, Harvinder S, Shaffie BM, Ramiza RR (2015). Anatomical variants of surgically important landmarks in the sphenoid sinus: a radiologic study in Southeast Asian patients. Surg Radiol Anat.

[10] Hewaidi G, Omami G (2008). Anatomic variation of sphenoid sinus and related structures in Libyan population: CT scan study. Libyan J Med.

[11] Tatreau JR, Patel MR, Shah RN (2010). Anatomical considerations for endoscopic endonasal skull base surgery in pediatric patients. Laryngoscope.

[12] Lu Y, Pan J, Qi S, Shi J, Zhang X, Wu K (2011). Pneumatization of the sphenoid sinus in Chinese: the differences from Caucasian and its application in the extended transsphenoidal approach. J Anat.

[13] Vemuri NV, Karanam LS, Manchikanti V, Dandamudi S, Puvvada SK, Vemuri VK (2017). Imaging review of cerebrospinal fluid leaks. Indian J Radiol Imaging.

[14] Unal B, Bademci G, Bilgili YK, Batay F, Avci E (2006). Risky anatomic variations of sphenoid sinus for surgery. Surg Radiol Anat.

[15] DeLano MC, Fun FY, Zinreich SJ (1996). Relationship of the optic nerve to the posterior paranasal sinuses: a CT anatomic study. AJNR Am J Neuroradiol.

